# Recent Advances in Polymers Bearing Activated Esters for the Synthesis of Glycopolymers by Postpolymerization Modification

**DOI:** 10.3390/polym16081100

**Published:** 2024-04-15

**Authors:** Tomonari Tanaka

**Affiliations:** Department of Biobased Materials Science, Graduate School of Science and Technology, Kyoto Institute of Technology, Matsugasaki, Sakyo-ku, Kyoto 606-8585, Japan; t-tanaka@kit.ac.jp

**Keywords:** glycopolymer, postpolymerization modification, activated ester, amidation, water-soluble, dual modification, synthesis in water

## Abstract

Glycopolymers are functional polymers with saccharide moieties on their side chains and are attractive candidates for biomaterials. Postpolymerization modification can be employed for the synthesis of glycopolymers. Activated esters are useful in various fields, including polymer chemistry and biochemistry, because of their high reactivity and ease of reaction. In particular, the formation of amide bonds caused by the reaction of activated esters with amino groups is of high synthetic chemical value owing to its high selectivity. It has been employed in the synthesis of various functional polymers, including glycopolymers. This paper reviews the recent advances in polymers bearing activated esters for the synthesis of glycopolymers by postpolymerization modification. The development of polymers bearing hydrophobic and hydrophilic activated esters is described. Although water-soluble activated esters are generally unstable and hydrolyzed in water, novel polymer backbones bearing water-soluble activated esters are stable and useful for postpolymerization modification for synthesizing glycopolymers in water. Dual postpolymerization modification can be employed to modify polymer side chains using two different molecules. Thiolactone and glycine propargyl esters on the polymer backbone are described as activated esters for dual postpolymerization modification.

## 1. Introduction

**Figure 1:**
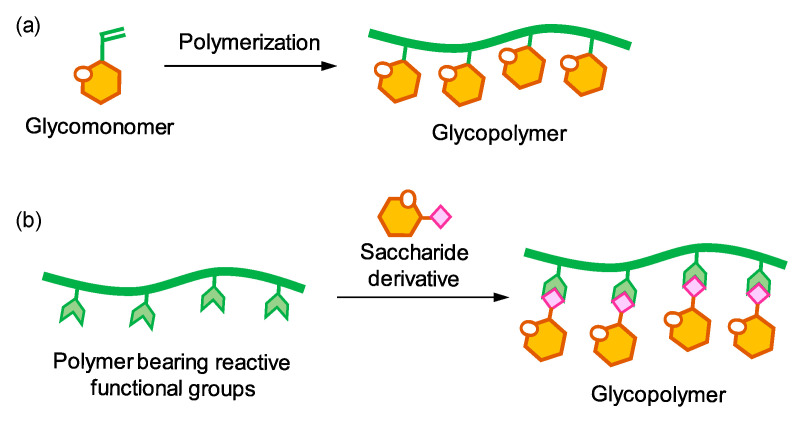
Synthesis methods for glycopolymers by (**a**) the polymerization of a glycomonomer and (**b**) postpolymerization modification.

Carbohydrates are one of the most abundant biomass resources and one of the most important biomolecules. Thus, carbohydrate-based functional polymers and materials are candidates for biomaterials [1,2,3]. Various carbohydrate polymers exist, such as natural polysaccharides, synthetic polysaccharides, and saccharide–synthetic polymer conjugates. Glycopolymers, synthetic polymers with pendant saccharide moieties on their side chains, are functional polymers and saccharide–synthetic polymer conjugates [4,5,6,7,8,9,10,11,12,13,14,15,16,17,18,19,20,21,22]. The most important characteristic of glycopolymers is their multivalent binding affinity, called the “glycocluster effect”, in which multiple saccharide moieties are densely packed around a polymer backbone, resulting in stronger binding to receptor biomolecules, such as lectins, viruses, and antibodies [23,24,25]. The synthesis of glycopolymers can be achieved via two processes: the polymerization of glycomonomers and the introduction of saccharide derivatives onto polymer side chains (Figure 1). The polymerization of a glycomonomer is advantageous in that the side chains of the resulting polymers bear saccharide moieties in a reliable and quantitative manner. In addition, various copolymers can be synthesized via copolymerization with other monomers. However, the steric hindrance of the saccharide moieties and the chemical structure of glycomonomers frequently affect the progress of polymerization. This is particularly relevant for large oligosaccharides comprising many monosaccharides. The introduction of saccharide derivatives onto polymer side chains is called “postpolymerization modification” [26,27,28,29,30]. In this method, glycopolymers are synthesized using pre-synthesized polymer backbones bearing reactive functional groups on the side chains through a reaction with saccharide derivatives that can react with and be introduced into the side chains. Sterically small saccharide derivatives, such as monosaccharide and disaccharide derivatives, can be introduced relatively easily. However, excess saccharide derivatives frequently need to be added to the reaction mixture to achieve a quantitatively high saccharide substitution. In addition, when the solubilities of polymer backbones and saccharide derivatives differ, the solvent should be carefully selected. An appropriate solvent that dissolves both the polymer backbone and the saccharide is required.

**Figure 2:**
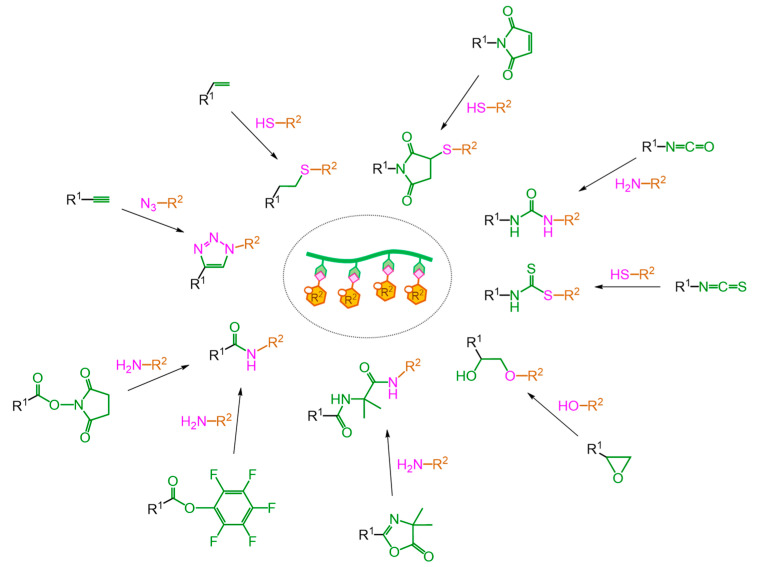
Reactions employed in postpolymerization modification.

Various reactions can be employed in postpolymerization modification (Figure 2). In particular, reactions with high reactivity and only a few side reactions are preferred. Reactions that proceed well under mild conditions, such as room temperature, neutral conditions, and without additives (catalysts and other agents), are particularly suitable for the introduction of biomolecules. Click chemistry, such as azide–alkyne cycloadditions (Huisgen cycloaddition) [31,32,33] and thiol-ene reactions [34,35,36,37], is frequently employed in postpolymerization modification. This is because, in most cases, click chemistry reactions do not produce byproducts, and they enable the facile linking of molecules. Huisgen cycloaddition has been employed to synthesize numerous glycomonomers and glycopolymers with the Haddleton, Gibson, Becer, and Miura groups, and many others [38,39,40,41,42,43,44,45,46,47,48,49,50,51,52,53,54]. The authors reported the synthesis of glycopolymers by Huisgen cycloaddition in the postpolymerization modification using glycosyl azides, which were directly synthesized from unprotected saccharides using a water-soluble dehydration condensation agent, 2-chloro-1,3-dimethylimidazolinium chloride in water [55,56,57,58,59,60,61]. Many reports exist on the investigation of the binding affinity of glycopolymers synthesized by Huisgen cycloaddition and other methods used against influenza viruses and their proteins [45,56,57,62,63]. Various other reactions have been used, such as those of maleimide and thiol; isocyanate and amine; isothiocyanate and thiol; epoxide and alcohol; azlactone and amine; and activated esters and amine. Multiple orthogonal reactions that do not interfere with each other can be combined to synthesize functional polymers via the postpolymerization modification of various combinations. This review summarizes the recent advances in polymers with pendant activated esters for the synthesis of glycopolymers via postpolymerization modification. In particular, the development of polymers bearing activated esters and dual modifiable functional groups is described.

## 2. Activated Esters in Postpolymerization Modification

**Scheme 1:**
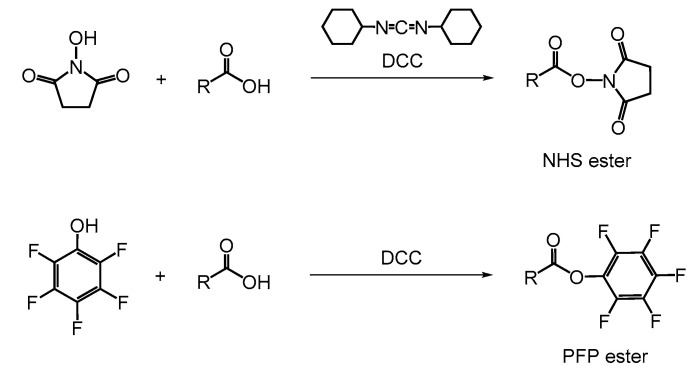
Syntheses of activated esters.

Activated esters are reactive esters that are more susceptible to nucleophilic attack than ordinary esters. The reactions of activated esters and amino groups form amide bonds under generally neutral conditions, resulting in the production of amide compounds. Base agents, such as tertiary amines, are typically added to promote amidation, although the reaction can be performed without a base agent. Activated esters can be converted into other esters via transesterification through a reaction with a hydroxy group under mostly basic conditions. Currently, hydrophobic activated esters, such as the *N*-hydroxysuccinimide (NHS) and pentafluorophenyl (PFP) esters, are representative activated esters in general use (Scheme 1). The syntheses of the derivatives of these activated esters are typically performed using the dehydration condensation reaction of NHS and pentafluorophenol, with carboxy-group-containing compounds using dehydration condensation agents, such as *N*,*N*′-dicyclohexylcarbodiimide (DCC) in a dehydrated organic solvent. Activated esters function as intermediates during the amidation and transesterification reactions of carboxylic compounds in dehydration condensation reactions. Activated ester intermediates are converted into amide and ester compounds by the nucleophilic attack of amino and hydroxy groups, respectively. Reactions using activated esters are typically employed in peptide synthesis using dehydration condensation agents.

**Figure 3:**
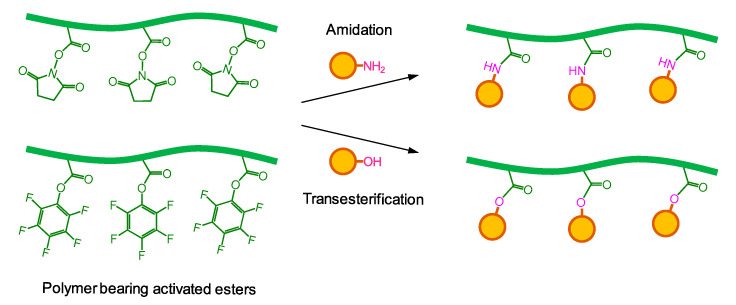
Postpolymerization modification using activated esters by amidation and transesterification.

**Scheme 2:**
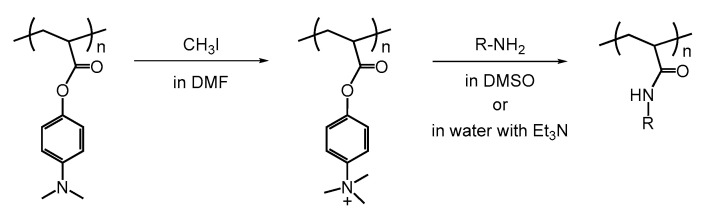
Activation and amidation of MAP-bearing polymer.

Polymers bearing activated esters were proposed as new synthetic compounds for preparing pharmacologically activated polymer–drug conjugates by Ringsdorf et al. in the 1970s [64]. Postpolymerization modification using activated esters has been widely employed to synthesize functional polymers, including glycopolymers, because one of the advantages of using activated esters is that they allow for the synthesis of a library of diverse polymers without disrupting key polymer parameters such as molecular weight. Numerous studies on postpolymerization modification using NHS and PFP esters have been reported (Figure 3), and several reviews exist [27,28,65]. The amidation of activated esters with amino compounds is frequently employed among the previously described reactions for postpolymerization modification because it demonstrates good reactivity even under neutral, open-to-air, aqueous, and catalyst-free conditions. The transesterification of activated esters with alcohol compounds has been employed in postpolymerization modification. Theato’s group reported the transesterification of the PFP ester on a polymer side chain [66]. Transesterification reactions on the side chain of a polyacrylate backbone using various alcohol compounds in the presence of dimethylaminopyridine (DMAP) in *N*,*N*-dimethylformamide (DMF) have been reported. Theato’s group reported polymers bearing salicylic-acid-based derivatives as less cytotoxic activated esters for postpolymerization modification [67,68]. Zhao’s group reported polymer backbones bearing 4-(dimethylamino)phenyl (MAP) groups as an activated ester [69]. The MAP group on a polyacrylate backbone does not react with an amino group; however, it can be activated by the addition of iodomethane in DMF and reacts with primary amino groups in dimethyl sulfoxide (DMSO) (Scheme 2). The amidation of a MAP-bearing polymer with amino acids in water can be performed in the presence of triethylamine (Et_3_N). The MAP group changes into an electron-withdrawing group from an electron-donating group via the modulation of reactivity. Owing to these reports, activated esters have been widely used in postpolymerization modification to synthesize functional polymers. In addition, the terminal modification of polymer backbones using activated esters has been reported for the introduction of functional groups, including saccharides [70,71,72,73].

## 3. Synthesis of Glycopolymers Using Polymers Bearing Activated Esters

Numerous postpolymerization modification techniques have been employed for the synthesis of polymeric molecules and functional materials. The aforementioned reactions with high reactivity and a few side reactions—such as the Huisgen cycloaddition, thiol-ene reactions, and the amidation of an activated ester with an amino compound—are frequently employed in postpolymerization modification. This is performed to synthesize functional polymers with various functional groups in their side chains. Several reviews have been published on postpolymerization modification using common hydrophobic activated esters, such as the NHS and PFP esters [27,28,65]. Similarly, the synthesis of glycopolymers has been widely reported. Most of the postpolymerization modifications using NHS and PFP esters are performed in organic solvents because activated esters are hydrophobic. Thus, polar aprotic organic solvents, such as DMSO and DMF, are typically used because of the solubility of the unprotected saccharide derivatives when glycopolymers are synthesized by postpolymerization modification using common hydrophobic activated esters and unprotected saccharide derivatives.

**Scheme 3:**
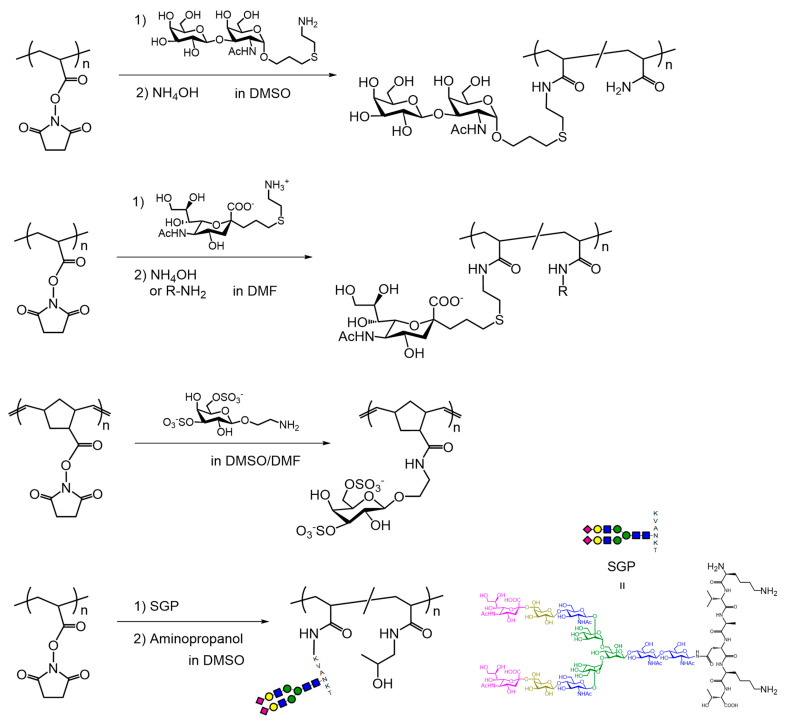
Synthesis of glycopolymers by postpolymerization modification using polymers bearing NHS esters.

**Scheme 4:**
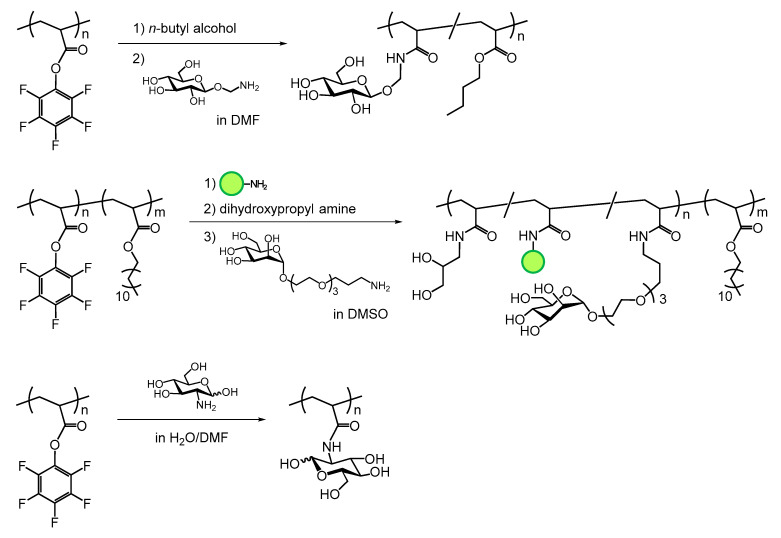
Synthesis of glycopolymers by postpolymerization modification using polymers bearing PFP esters.

Many researchers have reported the synthesis of glycopolymers by postpolymerization modification using activated esters, such as NHS [74,75,76,77] and PFP [78,79] esters on various synthetic polymer backbones, such as polyacrylamides, poly(metha)acrylates, and polynorbornenes (Scheme 3 and Scheme 4). Polymers bearing NHS and PFP esters have been reacted with amino-group-containing saccharide derivatives to synthesize glycopolymers in DMSO and DMF. The authors reported the synthesis of glycopolymers bearing sialyl-complex-type oligosaccharides using the NHS-bearing polymer and sialylglycopeptide (SGP) in DMSO [62]. The residual activated esters on the polymer side chain generally react with excess amino or alcohol compounds to remove the activated esters after amidation with saccharide derivatives. Amino sugars, such as glucosamine and galactosamine, which bear an amino group at the 2-position, have been used to synthesize glycopolymers [80,81,82,83]. In these cases, the amino sugars functioned as easy-to-use hydrophilic amino compounds to impart hydrophilicity on the resulting polymer products. The solvent in the amidation using glucosamine was DMF-containing water [80].

The combination of two different modification reactions on polymer side chains can broaden the range of applications. Gibson and Liu’s groups synthesized glycopolymers via the combination of amidation using the PFP ester and Huisgen cycloaddition with glycosyl azides [84,85]. Liu’s group synthesized glycopolymers via the combination of Huisgen cycloaddition with glycosyl azides and a thiol-ene reaction with glycosyl thiols [54]. Many examples exist for the synthesis of glycopolymers via postpolymerization modification using polymer backbones bearing activated esters and other reactive groups that cannot be depicted herein.

## 4. Synthesis of Glycopolymers Using Polymers Bearing Water-Soluble Activated Esters in Water

**Scheme 5:**
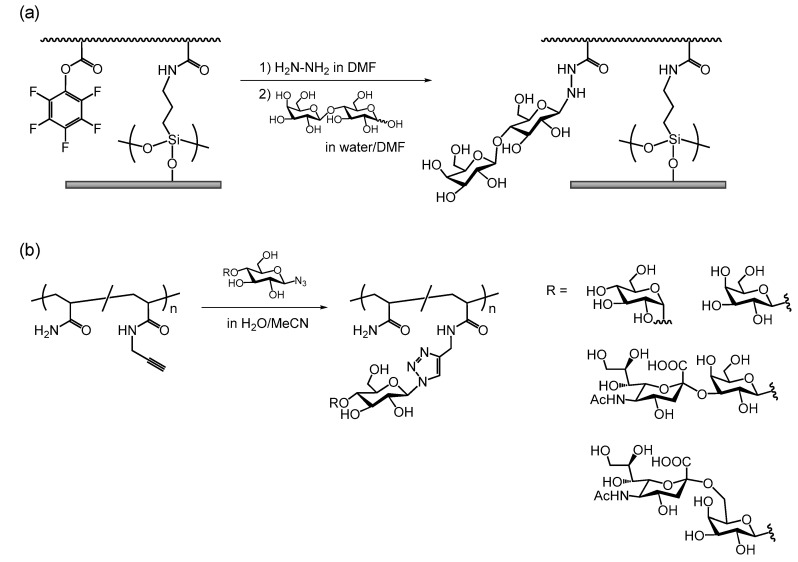
Postpolymerization modification under aqueous conditions.

Postpolymerization modification in water is advantageous for the synthesis of polymer–biomolecular conjugates, particularly conjugates with proteins and saccharides. Numerous reports of postpolymerization modification in organic solvents have been published, and a few reports have been published on the modification in aqueous media, such as a cosolvent with water and a water-soluble organic solvent. However, there are a few reports on postpolymerization modification in water, apart from the use of activated esters in postpolymerization modification. Chen et al. prepared glycopolymer-grafted silicon surfaces via postpolymerization modification using polymers bearing PFP esters, which reacted with hydrazide on the surface in DMF and subsequently reacted with free lactose in DMF-containing water in the presence of aniline (Scheme 5a) [86]. As an example of the use of other materials apart from activated esters, Nagao et al. synthesized glycopolymers via Huisgen cycloaddition using glycosyl azides and polymers bearing alkynyl groups in aqueous media (acetonitrile-containing water) (Scheme 5b) [45]. In this case, a water-mixable organic solvent, acetonitrile, was used because of the low water solubility of the polymer backbone and the additive, tris(benzyltriazolylmethyl)amine, used as the ligand for a copper(I) catalyst. Incidentally, the water-soluble ligand tris(3-hydroxypropyltriazolylmethyl)amine is commercially available [87,88]. Moreover, there are reports on postpolymerization modification using random and block copolymers bearing activated esters and hydrophilic moieties to synthesize polymers with pendant peptides and nanohydrogel particles [89,90,91].

**Figure 4:**
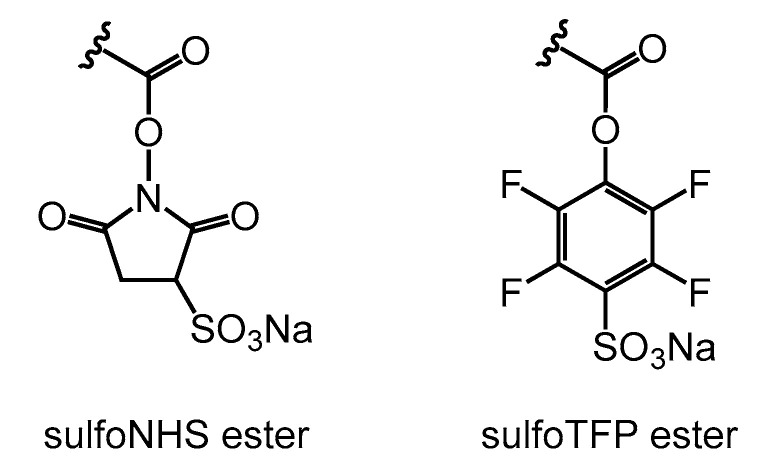
Chemical structures of water-soluble activated esters.

**Figure 5:**
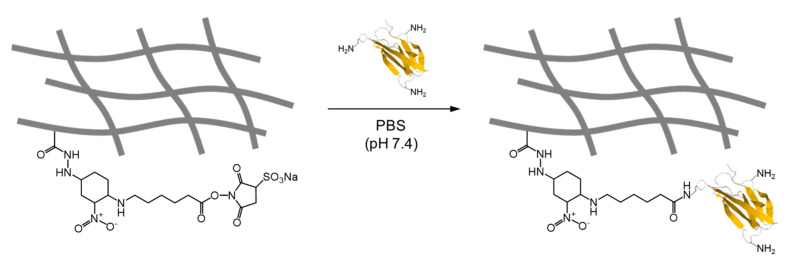
Immobilization of proteins on polymer hydrogels using a water-soluble activated ester.

**Scheme 6:**
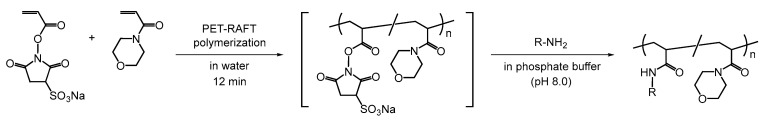
Synthesis of sulfoNHS-bearing polymers and postpolymerization modification in water.

Postpolymerization modification is employed in chemical biology in the labeling and immobilization of biomolecules, such as proteins and antibodies [92,93,94,95]. Activated esters are also applied for the synthesis of the conjugates of biomacromolecules and synthetic polymers, such as protein–polymer conjugates [96]. The use of activated esters, such as NHS and PFP esters, under aqueous conditions is limited to a mixture of water with water-mixable organic solvents because they are generally hydrophobic and water-insoluble. Thus, water-soluble activated esters—such as *N*-hydroxysulfosuccinimide (sulfoNHS) [97,98,99,100,101] and 4-sulfo-2,3,5,6-tetrafluorophenyl (sulfoTFP) [102,103] esters, in which a sulfate group has been introduced into the NHS and PFP moieties—were developed to endow them with water solubility for use in water (Figure 4). Water-soluble activated esters, such as sulfoNHS and sulfoTFP esters, enable the synthesis of functional polymers by postpolymerization modification in water. Poellmann et al. reported the use of the sulfoNHS ester for the immobilization of fibronectin on polyacrylamide hydrogels in an aqueous buffer media (Figure 5) [104]. Trappmann et al. reported the use of the sulfoNHS ester to attach collagen to polyacrylamide hydrogels for the investigation of stem-cell fate in the extracellular matrix [105]. However, there are a few reports on synthetic polymers bearing water-soluble activated esters. Niu et al. reported that a monomer bearing a water-soluble activated ester, an acrylate derivative bearing the sulfoNHS ester, was extremely unstable in water with a half-life of 1 h (Scheme 6) [106]. Thus, photoinduced electron transfer–reversible addition–fragmentation chain transfer (PET-RAFT) polymerization was performed in an extremely short time (12 min) in water to synthesize a polymer backbone bearing sulfoNHS esters by minimizing the degradation of sulfoNHS ester in water. The resulting polymer bearing sulfoNHS esters was expected to exhibit low stability in water, similar to the monomer. A subsequent postpolymerization modification with L-phenylalanine, 5-[(2-aminoethyl)amino]-naphthalene-1-sulfonic acid sodium salt, and bovine serum albumin was performed without isolating the sulfoNHS-bearing polymers in aqueous buffer.

**Scheme 7:**
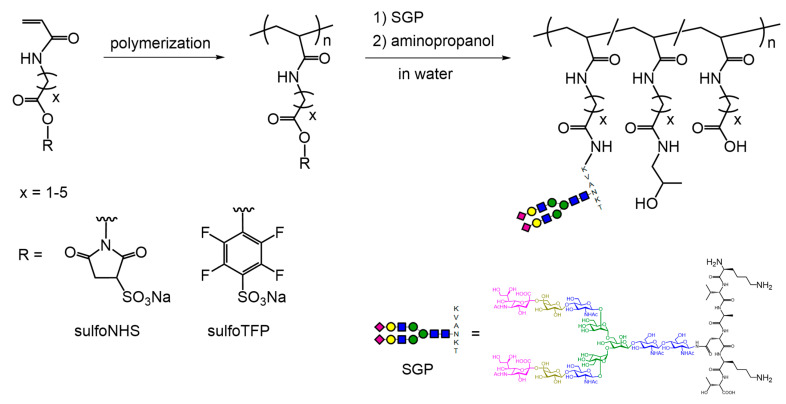
Synthesis of polymers bearing sulfoNHS and sulfoTFP esters and synthesis of glycopolymers in water by postpolymerization modification.

The development of polymers bearing water-soluble activated esters that are stable and easy to use in water facilitates the implementation of reactions in only water and reduces the limitations of postpolymerization modification. The author and coworkers recently developed water-stable monomers bearing a water-soluble activated ester and their polymers for the synthesis of glycopolymers by postpolymerization modification in water (Scheme 7). An acrylamide derivative bearing a sulfoNHS ester was synthesized by the dehydration condensation reaction of acrylamide alkanoic acid with sodium sulfoNHS using DCC. Similarly, a monomer bearing sulfoTFP was synthesized via a dehydration condensation reaction with sodium 2,3,5,6-tetrafluoro-4-hydroxybenzenesulfonate. These water-soluble ester-bearing monomers with a methylene chain between the amide and activated ester groups exhibited higher stability in water than the aforementioned water-soluble activated ester-bearing monomer, in which the activated ester was directly attached to the monomer moiety. The half-life of the monomer bearing the sulfoNHS ester with a methylene chain (x = 5) was 16 h in water [107]. In the case of monomers bearing sulfoTFP esters, monomers with different methylene chain lengths (x = 1–5) were synthesized [108]. The stability in water of monomers with longer methylene chains was significantly better than that of monomers with shorter methylene chains, particularly when the methylene chain length was x = 3 or more. When the methylene chain length was x = 5, the half-life in water increased to 50 h. All the polymers obtained by the radical polymerization of water-soluble activated ester-bearing monomers could be isolated and purified by reprecipitation. The stability of the water-soluble activated esters in the side chains of the polymers in water exceeded that of the corresponding monomers. In particular, when the methylene chain length was x = 5, the 80% residual times of the sulfoNHS and sulfoTFP esters in the polymer side chain were 9 and 90 h, respectively, showing a significant improvement in their stability in water. Thus, postpolymerization modification using polymers bearing sulfoNHS or sulfoTFP esters in water was performed to synthesize glycopolymers bearing SGP in water (Scheme 7) [108,109]. The polymerization of water-soluble activated ester-bearing monomers and subsequent postpolymerization modification can be conducted in a one-pot process. PET-RAFT polymerization, which can be performed as controlled radical polymerization in an open-air atmosphere, has been conducted in water. Postpolymerization modification has been performed without isolating the resulting polymers bearing water-soluble activated esters, thereby achieving the one-pot synthesis of glycopolymers in water in an open-air atmosphere [110].

**Scheme 8:**
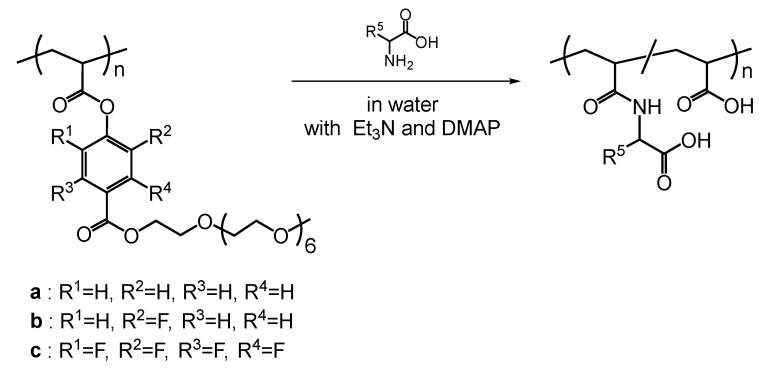
Postpolymerization modification using polymers bearing fluorobenzene-based activated esters in water.

Zhao’s group reported monomers bearing water-soluble activated esters, which were fluorobenzene derivatives with oligoethylene glycol and their polymers (Scheme 8) [111]. These monomers and polymers included compounds with extremely high stability in water (they hardly decompose in water). However, their amidation reactivity with amino groups was low because of their high stability in water. During the postpolymerization modification using excess amounts of amino acids, the degree of substitution reached 10–90% in the presence of Et_3_N and DMAP under basic conditions. The introduction of papain, a protease, into the polymer side chain has been reported during postpolymerization modification using this polymer in water. Similarly, amino-acid-bearing polymers have been synthesized using activated esters, such as fluorobenzene esters in DMF and water-containing DMSO [112,113].

## 5. Dual Postpolymerization Modification

**Figure 6:**
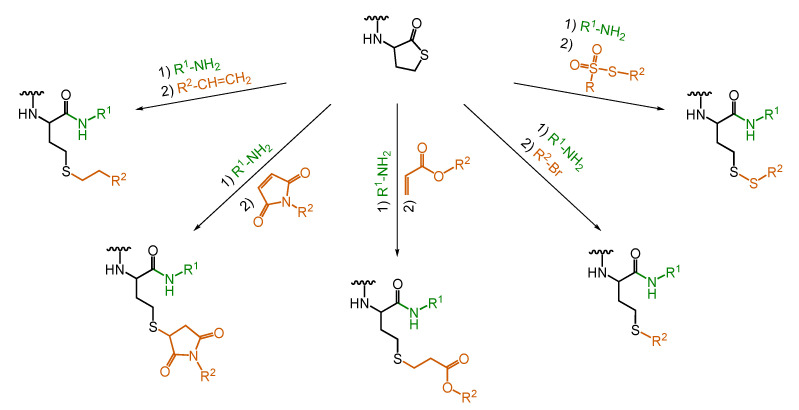
Dual modification reactions using thiolactone.

Modification via two different functional groups in polymer side chains using postpolymerization modification is attractive for synthesizing diverse and highly functional polymers. Certain sequential multifunctionalizations for a single polymer side chain have been reported [29]. Thiolactone is a dual-functionalized group. It reacts with an amino group via a ring-opening reaction to form amide bonds as the first modification (Figure 6) [114]. After the ring-opening reaction, the thiol group produced from thiolactone allows for a variety of second modification reactions in a sequential manner.

**Scheme 9:**
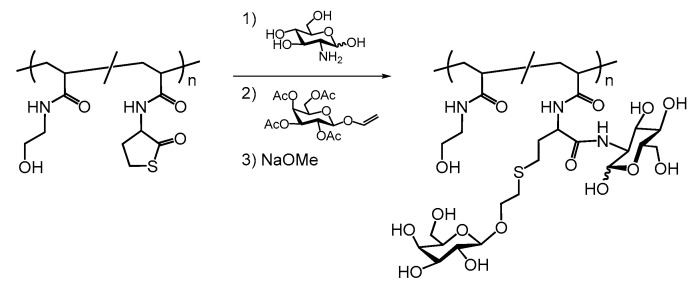
Synthesis of glycopolymers by dual modification using thiolactone-bearing polymers.

The occurrence of two different reactions in a one-pot manner by reacting thiol with alkene (thiol-ene reaction), the formation of a thiourea bond by reaction with an isocyanate group, and the formation of a disulfide bond with another thiol derivative are advantageous. Prez’s group reported a one-pot reaction, an amidation process with various amino compounds, and a subsequent thiol-ene reaction with acrylate derivatives using the copolymers of polyacrylamide derivatives bearing thiolactone groups and poly(*N*-isopropylacrylamide) (PNIPAM) [115]. The resulting PNIPAM copolymers had lower critical solution temperatures. This was accompanied by the ability to undergo two types of modification reactions in a one-pot manner, i.e., without isolating the first product. Gibson’s group synthesized double-modified glycopolymers bearing two monosaccharides, galactose and glucosamine, as a mimic of ganglioside GM1 glycan (Scheme 9) [116]. Many other syntheses of functional polymers using thiolactone have been reported [117,118,119,120]. Azlactone was similarly used for dual postpolymerization modification. Moore’s group synthesized protein–polymer conjugates by modifying some of the azlactones in polymer side chains with tetraethylene glycol to render them hydrophilic and modifying the protein via amidation in a buffer solution containing 15% DMSO and the remaining azlactone [121]. A copper-catalyzed multicomponent reaction of alkynes with amines and sulfonyl azides was reported as a dual postpolymerization modification [122].

**Scheme 10:**
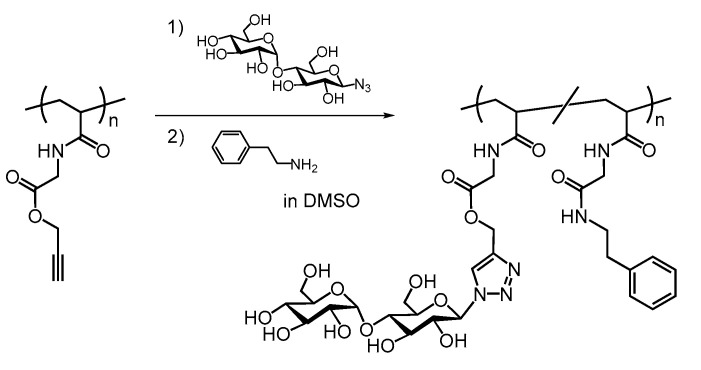
One-pot and dual postpolymerization modification using a polymer bearing glycine propargyl esters.

Recently, Tanaka’s group reported that the propargyl ester of glycine can be used in amidation reactions as an activated ester [123]. Polyamine-selective reactive probes were synthesized using the glycine propargyl ester and applied to selective cancer-cell imaging studies [124]. The amidation of the glycine propargyl ester exhibited good reactivity with primary amines but did not proceed with amino compounds with carboxy groups, such as amino acids and their ester derivatives, aromatic amino compounds, and secondary amines. The authors synthesized polyacrylamide polymer backbones bearing glycine propargyl esters [125]. Dual postpolymerization modification using polymers bearing glycine propargyl esters afforded glycopolymers in a one-pot manner combined with Huisgen cycloaddition with a glycosyl azide and amidation with an amino compound on glycine propargyl esters (Scheme 10). In the aforementioned study, the amidation ratio of glycine propargyl esters on the polymer side chain exceeded that of the monomeric glycine propargyl ester. Amino groups with low nucleophilicity, including secondary amines, did not react with the glycine propargyl ester on the polymer side chains. The amidation ratio of the copolymers bearing glycine propargyl esters with a high substitution ratio exceeded that of the polymer with a low glycine propargyl ester substitution ratio. It has been suggested that the amidation of the glycine propargyl ester is promoted by the increased nucleophilicity of the amino groups caused by the hydrogen bonding of neighboring propargyl esters on the side chain acting as a base. Through postpolymerization modification using a polymer bearing glycine propargyl esters, a part of the glycine propargyl esters on the polymer side chains was modified via Huisgen cycloaddition with maltosyl azide. This was then followed by amidation with hydrophobic phenylethylamine for the remaining glycine propargylic ester. The resulting glycopolymer formed aggregates with saccharide moieties presented as a shell in water.

## 6. Conclusions

Activated esters have been used for modification reactions in various fields, including polymer chemistry and biochemistry, because of their high reactivity and ease of reaction. In particular, the reaction of activated esters with amino groups is highly valuable in synthetic chemistry because the reaction proceeds well even in water under relatively mild conditions. Monomers bearing a water-soluble activated ester and their polymers, which improve the low stability of water-soluble activated esters in water, broaden the possibility of synthesizing hydrophilic functional polymers, such as glycopolymers and protein–polymer conjugates, by postpolymerization modification in water. Although a few side reactions occur, including the hydrolysis of activated esters, the resulting amide bond is attractive and widely used because of its highly stable and simple chemical structure. Dual postpolymerization modification holds great potential for the synthesis of diverse and highly functional polymer materials. Thus, we envision that activated esters and their reactions will be developed, leading to the development of further applications in the future.

## References

[1] Su L., Feng Y., Wei K., Xu X., Liu R., Chen G. (2021). Carbohydrate-Based Macromolecular Biomaterials. Chem. Rev..

[2] Chen G. (2021). The Past Ten Years of Carbohydrate Polymers in ACS Macro Letters. ACS Macro Lett..

[3] Delbianco M., Bharate P., Varela-Aramburu S., Seeberger P.H. (2016). Carbohydrates in Supramolecular Chemistry. Chem. Rev..

[4] Le Droumaguet B., Nicolas J. (2010). Recent Advances in the Design of Bioconjugates from Controlled/Living Radical Polymerization. Polym. Chem..

[5] Vázquez-Dorbatt V., Lee J., Lin E., Maynard H.D. (2012). Synthesis of Glycopolymers by Controlled Radical Polymerization Techniques and Their Applications. ChemBioChem.

[6] Miura Y. (2012). Design and Synthesis of Well-Defined Glycopolymers for the Control of Biological Functionalities. Polym. J..

[7] Sunasee R., Narain R. (2013). Glycopolymers and Glyco-Nanoparticles in Biomolecular Recognition Processes and Vaccine Development. Macromol. Biosci..

[8] Ahmed M., Wattanaarsakit P., Narain R. (2013). Recent Advances in the Preparation of Glycopolymer Bioconjugates. Eur. Polym. J..

[9] Miura Y., Hoshino Y., Seto H. (2016). Glycopolymer Nanobiotechnology. Chem. Rev..

[10] Abdouni Y., Yilmaz G., Becer C.R. (2017). Sequence and Architectural Control in Glycopolymer Synthesis. Macromol. Rapid Commun..

[11] Pramudya I., Chung H. (2019). Recent Progress of Glycopolymer Synthesis for Biomedical Applications. Biomater. Sci..

[12] Ma Z., Zhu X.X. (2019). Copolymers Containing Carbohydrates and Other Biomolecules: Design, Synthesis and Applications. J. Mater. Chem. B.

[13] Stenzel M.H. (2022). Glycopolymers for Drug Delivery: Opportunities and Challenges. Macromolecules.

[14] Bianculli R.H., Mase J.D., Schulz M.D. (2020). Antiviral Polymers: Past Approaches and Future Possibilities. Macromolecules.

[15] Kim Y., Hyun J.Y., Shin I. (2021). Multivalent Glycans for Biological and Biomedical Applications. Chem. Soc. Rev..

[16] Richards S.-J., Gibson M.I. (2021). Toward Glycomaterials with Selectivity as Well as Affinity. JACS Au.

[17] Zhao T., Terracciano R., Becker J., Monaco A., Yilmaz G., Becer C.R. (2022). Hierarchy of Complex Glycomacromolecules: From Controlled Topologies to Biomedical Applications. Biomacromolecules.

[18] Bhattacharya K., Kalita U., Singha N.K. (2022). Tailor-Made Glycopolymers via Reversible Deactivation Radical Polymerization: Design, Properties and Applications. Polym. Chem..

[19] Wang J., Zhou J., Ding Y., Hu X., Chen Y. (2023). Glycopolymers Based on Carbohydrate or Vinyl Backbones and Their Biomedical Applications. Polym. Chem..

[20] Martínez-Bailén M., Rojo J., Ramos-Soriano J. (2023). Multivalent Glycosystems for Human Lectins. Chem. Soc. Rev..

[21] Mei R., Heng X., Liu X., Chen G. (2023). Glycopolymers for Antibacterial and Antiviral Applications. Molecules.

[22] Nagao M., Matsumoto H., Miura Y. (2023). Design of Glycopolymers for Controlling the Interactions with Lectins. Chem. Asian J..

[23] Lee Y.C., Lee R.T. (1995). Carbohydrate-Protein Interactions: Basis of Glycobiology. Acc. Chem. Res..

[24] Mammen M., Choi S.K., Whitesides G.M. (1998). Polyvalent Interactions in Biological Systems: Implications for Design and Use of Multivalent Ligands and Inhibitors. Angew. Chem. Int. Ed..

[25] Lundquist J.J., Toone E.J. (2002). The Cluster Glycoside Effect. Chem. Rev..

[26] Gauthier M.A., Gibson M.I., Klok H.A. (2009). Synthesis of Functional Polymers by Post-Polymerization Modification. Angew. Chem. Int. Ed..

[27] Das A., Theato P. (2016). Activated Ester Containing Polymers: Opportunities and Challenges for the Design of Functional Macromolecules. Chem. Rev..

[28] Blasco E., Sims M.B., Goldmann A.S., Sumerlin B.S., Barner-Kowollik C. (2017). *50th Anniversary Perspective*: Polymer Functionalization. Macromolecules.

[29] Kubo T., Easterling C.P., Olson R.A., Sumerlin B.S. (2018). Synthesis of Multifunctional Homopolymers via Sequential Post-Polymerization Reactions. Polym. Chem..

[30] Chen X., Michinobu T. (2022). Postpolymerization Modification: A Powerful Tool for the Synthesis and Function Tuning of Stimuli-Responsive Polymers. Macromol. Chem. Phys..

[31] Kolb H.C., Finn M.G., Sharpless K.B. (2001). Click Chemistry: Diverse Chemical Function from a Few Good Reactions. Angew. Chem. Int. Ed..

[32] Dondoni A. (2007). Triazole: The Keystone in Glycosylated Molecular Architectures Constructed by a Click Reaction. Chem. Asian J..

[33] Witczak Z.J., Bielski R. (2013). Click Chemistry in Glycoscience.

[34] Hoyle C.E., Bowman C.N. (2010). Thiol–Ene Click Chemistry. Angew. Chem. Int. Ed..

[35] Lowe A.B. (2010). Thiol-Ene “Click” Reactions and Recent Applications in Polymer and Materials Synthesis. Polym. Chem..

[36] Dondoni A., Marra A. (2012). Recent Applications of Thiol–Ene Coupling as a Click Process for Glycoconjugation. Chem. Soc. Rev..

[37] Ahangarpour M., Kavianinia I., Harris P.W.R., Brimble M.A. (2021). Photo-Induced Radical Thiol–Ene Chemistry: A Versatile Toolbox for Peptide-Based Drug Design. Chem. Soc. Rev..

[38] Vinson N., Gou Y.Z., Becer C.R., Haddleton D.M., Gibson M.I. (2011). Optimised “click” Synthesis of Glycopolymers with Mono/Di- and Trisaccharides. Polym. Chem..

[39] Slavin S., Burns J., Haddleton D.M., Becer C.R. (2011). Synthesis of Glycopolymers via Click Reactions. Eur. Polym. J..

[40] Zhang Q., Collins J., Anastasaki A., Wallis R., Mitchell D.A., Becer C.R., Haddleton D.M. (2013). Sequence-Controlled Multi-Block Glycopolymers to Inhibit DC-SIGN-gp120 Binding. Angew. Chem. Int. Ed..

[41] Gou Y., Geng J., Richards S., Burns J., Remzi Becer C., Haddleton D.M. (2013). A Detailed Study on Understanding Glycopolymer Library and Con A Interactions. J. Polym. Sci. A Polym. Chem..

[42] Zhang Q., Wilson P., Anastasaki A., McHale R., Haddleton D.M. (2014). Synthesis and Aggregation of Double Hydrophilic Diblock Glycopolymers via Aqueous SET-LRP. ACS Macro Lett..

[43] Lu J., Zhang W., Richards S.-J., Gibson M.I., Chen G. (2014). Glycopolymer-Coated Gold Nanorods Synthesised by a One Pot Copper(0) Catalyzed Tandem RAFT/Click Reaction. Polym. Chem..

[44] Collis D.W.P., Yilmaz G., Yuan Y., Monaco A., Ochbaum G., Shi Y., O’Malley C., Uzunova V., Napier R., Bitton R. (2021). Hyaluronan (HA)-Inspired Glycopolymers as Molecular Tools for Studying HA Functions. RSC Chem. Biol..

[45] Nagao M., Kurebayashi Y., Seto H., Tanaka T., Takahashi T., Suzuki T., Hoshino Y., Miura Y. (2016). Synthesis of Well-Controlled Glycopolymers Bearing Oligosaccharides and Their Interactions with Influenza Viruses. Polym. J..

[46] Jono K., Nagao M., Oh T., Sonoda S., Hoshino Y., Miura Y. (2018). Controlling the Lectin Recognition of Glycopolymers via Distance Arrangement of Sugar Blocks. Chem. Commun..

[47] Terada Y., Hoshino Y., Miura Y. (2019). Glycopolymers Mimicking GM1 Gangliosides: Cooperativity of Galactose and Neuraminic Acid for Cholera Toxin Recognition. Chem. Asian J..

[48] Nagao M., Matsubara T., Hoshino Y., Sato T., Miura Y. (2019). Topological Design of Star Glycopolymers for Controlling the Interaction with the Influenza Virus. Bioconjug. Chem..

[49] Kichize M., Nagao M., Hoshino Y., Miura Y. (2020). Multi-Block and Sequence-Controlled Polymerization of Glycopolymers, and Interaction with Lectin. Eur. Polym. J..

[50] Nagao M., Uemura T., Horiuchi T., Hoshino Y., Miura Y. (2021). Screening of a Glycopolymer Library for GM1 Mimetics Synthesized by the “Carbohydrate Module Method”. Chem. Commun..

[51] Nagao M., Kichize M., Hoshino Y., Miura Y. (2021). Influence of Monomer Structures for Polymeric Multivalent Ligands: Consideration of the Molecular Mobility of Glycopolymers. Biomacromolecules.

[52] Ishida T., Nagao M., Oh T., Mori T., Hoshino Y., Miura Y. (2022). Synthesis of Glycopolymers Carrying 3′-Sialyllactose for Suppressing Inflammatory Reaction via Siglec-E. Chem. Lett..

[53] Malkoch M., Thibault R.J., Drockenmuller E., Messerschmidt M., Voit B., Russell T.P., Hawker C.J. (2005). Orthogonal Approaches to the Simultaneous and Cascade Functionalization of Macromolecules Using Click Chemistry. J. Am. Chem. Soc..

[54] Liu M., Wang X., Miao D., Wang C., Deng W. (2020). Synthesis of Well-Defined Heteroglycopolymers via Combining Sequential Click Reactions and PPM: The Effects of Linker and Heterogeneity on Con a Binding. Polym. Chem..

[55] Tanaka T., Nagai H., Noguchi M., Kobayashi A., Shoda S. (2009). One-Step Conversion of Unprotected Sugars to Beta-Glycosyl Azides Using 2-Chloroimidazolinium Salt in Aqueous Solution. Chem. Commun..

[56] Tanaka T., Ishitani H., Miura Y., Oishi K., Takahashi T., Suzuki T., Shoda S., Kimura Y. (2014). Protecting-Group-Free Synthesis of Glycopolymers Bearing Sialyloligosaccharide and Their High Binding with the Influenza Virus. ACS Macro Lett..

[57] Tanaka T., Zhou Y., Tamoto C., Kurebayashi Y., Takahashi T., Suzuki T. (2017). An α2,3-Linked Sialylglycopolymer as a Multivalent Glycoligand against Avian and Human Influenza Viruses. J. Appl. Glycosci..

[58] Tanaka T., Fukumoto H., Ishitani H. (2017). Synthesis of Glycopolymer Gold Nanoparticles Decorated with Oligosaccharides via a Protecting-Group-Free Process and Their Specific Recognition by Lectins. Trans. Mater. Res. Soc. Jpn..

[59] Tanaka T., Okamoto M. (2018). Reversible Temperature-Responsive and Lectin-Recognizing Glycosylated Block Copolymers Synthesized by RAFT Polymerization. Polym. J..

[60] Tanaka T., Okamoto M. (2018). Lectin and Temperature Dual-Responsive Glycosylated Block Copolymers Synthesized by Consecutive RAFT Polymerization Reactions. Bull. Chem. Soc. Jpn..

[61] Tsuji S., Aoki T., Ushio S., Tanaka T. (2020). Synthesis and Aggregation Behavior of Temperature- and pH-Responsive Glycopolymers as Sugar-Displaying Conjugates. Polymers.

[62] Tanaka T., Nakashima K., Tsuji S., Han X., Zhao J., Honda Y., Sakakibara K., Kurebayashi Y., Takahashi T., Suzuki T. (2019). Controlled Synthesis of Glycopolymers with Pendant Complex-Type Sialylglycopeptides and Their Binding Affinity with a Lectin and an Influenza Virus. Polym. Chem..

[63] Li G., Ma W., Mo J., Cheng B., Shoda S., Zhou D., Ye X.-S. (2021). Influenza Virus Precision Diagnosis and Continuous Purification Enabled by Neuraminidase-Resistant Glycopolymer-Coated Microbeads. ACS Appl. Mater. Interfaces.

[64] Batz H., Franzmann G., Ringsdorf H. (1972). Model Reactions for Synthesis of Pharmacologically Active Polymers by Way of Monomeric and Polymeric Reactive Esters. Angew. Chem. Int. Ed. Eng..

[65] Theato P. (2008). Synthesis of Well-Defined Polymeric Activated Esters. J. Polym. Sci. A Polym. Chem..

[66] Das A., Theato P. (2015). Multifaceted Synthetic Route to Functional Polyacrylates by Transesterification of Poly(Pentafluorophenyl Acrylates). Macromolecules.

[67] He L., Szameit K., Zhao H., Hahn U., Theato P. (2014). Postpolymerization Modification Using Less Cytotoxic Activated Ester Polymers for the Synthesis of Biological Active Polymers. Biomacromolecules.

[68] Zhang X., Li Z., Lin S., Theato P. (2020). Fibrous Materials Based on Polymeric Salicyl Active Esters as Efficient Adsorbents for Selective Removal of Anionic Dye. ACS Appl. Mater. Interfaces.

[69] Lang X., Xu Z., Li Q., Yuan L., Thumu U., Zhao H. (2022). Modulating the Reactivity of Polymer with Pendant Ester Groups by Methylation Reaction for Preparing Functional Polymers. Polym. Chem..

[70] Won S., Hindmarsh S., Gibson M.I. (2018). Triggerable Multivalent Glyconanoparticles for Probing Carbohydrate–Carbohydrate Interactions. ACS Macro Lett..

[71] Richards S.-J., Baker A.N., Walker M., Gibson M.I. (2020). Polymer-Stabilized Sialylated Nanoparticles: Synthesis, Optimization, and Differential Binding to Influenza Hemagglutinins. Biomacromolecules.

[72] Baker A.N., Richards S.-J., Pandey S., Guy C.S., Ahmad A., Hasan M., Biggs C.I., Georgiou P.G., Zwetsloot A.J., Straube A. (2021). Glycan-Based Flow-Through Device for the Detection of SARS-COV-2. ACS Sens..

[73] Georgiou P.G., Guy C.S., Hasan M., Ahmad A., Richards S.-J., Baker A.N., Thakkar N.V., Walker M., Pandey S., Anderson N.R. (2022). Plasmonic Detection of SARS-CoV-2 Spike Protein with Polymer-Stabilized Glycosylated Gold Nanorods. ACS Macro Lett..

[74] Mammen M., Dahmann G., Whitesides G.M. (1995). Effective Inhibitors of Hemagglutination by Influenza Virus Synthesized from Polymers Having Active Ester Groups. Insight into Mechanism of Inhibition. J. Med. Chem..

[75] Sigal G.B., Mammen M., Dahmann G., Whitesides G.M. (1996). Polyacrylamides Bearing Pendant α-Sialoside Groups Strongly Inhibit Agglutination of Erythrocytes by Influenza Virus: The Strong Inhibition Reflects Enhanced Binding through Cooperative Polyvalent Interactions. J. Am. Chem. Soc..

[76] Baek M.-G., Roy R. (2001). Relative Lectin Binding Properties of T-Antigen-Containing Glycopolymers: Copolymerization of N-Acryloylated T-Antigen Monomer vs. Graft Conjugation of Aminated T-Antigen Ligands onto Poly(N-Acryloxysuccinimide). Macromol. Biosci..

[77] Gordon E.J., Gestwicki J.E., Strong L.E., Kiessling L.L. (2000). Synthesis of End-Labeled Multivalent Ligands for Exploring Cell-Surface-Receptor–Ligand Interactions. Chem. Biol..

[78] Eissa A.M., Abdulkarim A., Sharples G.J., Cameron N.R. (2016). Glycosylated Nanoparticles as Efficient Antimicrobial Delivery Agents. Biomacromolecules.

[79] Scherer M., Kappel C., Mohr N., Fischer K., Heller P., Forst R., Depoix F., Bros M., Zentel R. (2016). Functionalization of Active Ester-Based Polymersomes for Enhanced Cell Uptake and Stimuli-Responsive Cargo Release. Biomacromolecules.

[80] Boyer C., Davis T.P. (2009). One-Pot Synthesis and Biofunctionalization of Glycopolymers via RAFT Polymerization and Thiol-Ene Reactions. Chem. Commun..

[81] Boyer C., Whittaker M., Davis T.P. (2011). Synthesis and Postfunctionalization of Well-defined Star Polymers via “Double” Click Chemistry. J. Polym. Sci. A Polym. Chem..

[82] Utama R.H., Jiang Y., Zetterlund P.B., Stenzel M.H. (2015). Biocompatible Glycopolymer Nanocapsules via Inverse Miniemulsion Periphery RAFT Polymerization for the Delivery of Gemcitabine. Biomacromolecules.

[83] Gaballa H., Theato P. (2019). Glucose-Responsive Polymeric Micelles via Boronic Acid–Diol Complexation for Insulin Delivery at Neutral pH. Biomacromolecules.

[84] Richards S., Jones M.W., Hunaban M., Haddleton D.M., Gibson M.I. (2012). Probing Bacterial-Toxin Inhibition with Synthetic Glycopolymers Prepared by Tandem Post-Polymerization Modification: Role of Linker Length and Carbohydrate Density. Angew. Chem. Int. Ed..

[85] Liu M., Miao D., Wang X., Wang C., Deng W. (2020). Precise Synthesis of Heterogeneous Glycopolymers with Well-defined Saccharide Motifs in the Side Chain via Post-polymerization Modification and Recognition with Lectin. J. Polym. Sci..

[86] Chen L., Leman D., Williams C.R., Durie K., Krause D.C., Locklin J. (2017). Versatile Methodology for Glycosurfaces: Direct Ligation of Nonderivatized Reducing Saccharides to Poly(Pentafluorophenyl Acrylate) Grafted Surfaces via Hydrazide Conjugation. Langmuir.

[87] Hong V., Steinmetz N.F., Manchester M., Finn M.G. (2010). Labeling Live Cells by Copper-Catalyzed Alkyne–Azide Click Chemistry. Bioconjug. Chem..

[88] Kennedy D.C., McKay C.S., Legault M.C.B., Danielson D.C., Blake J.A., Pegoraro A.F., Stolow A., Mester Z., Pezacki J.P. (2011). Cellular Consequences of Copper Complexes Used to Catalyze Bioorthogonal Click Reactions. J. Am. Chem. Soc..

[89] Moghaddam M.J., Matsuda T. (1993). Molecular Design of Three-dimensional Artificial Extracellular Matrix: Photosensitive Polymers Containing Cell Adhesive Peptide. J. Polym. Sci. A Polym. Chem..

[90] Nuhn L., Gietzen S., Mohr K., Fischer K., Toh K., Miyata K., Matsumoto Y., Kataoka K., Schmidt M., Zentel R. (2014). Aggregation Behavior of Cationic Nanohydrogel Particles in Human Blood Serum. Biomacromolecules.

[91] Leber N., Nuhn L., Zentel R. (2017). Cationic Nanohydrogel Particles for Therapeutic Oligonucleotide Delivery. Macromol. Biosci..

[92] Spicer C.D., Davis B.G. (2014). Selective Chemical Protein Modification. Nat. Commun..

[93] Boutureira O., Bernardes G.J.L. (2015). Advances in Chemical Protein Modification. Chem. Rev..

[94] Chari R.V.J., Miller M.L., Widdison W.C. (2014). Antibody–Drug Conjugates: An Emerging Concept in Cancer Therapy. Angew. Chem. Int. Ed..

[95] Messina M.S., Messina K.M.M., Bhattacharya A., Montgomery H.R., Maynard H.D. (2020). Preparation of Biomolecule-Polymer Conjugates by Grafting-from Using ATRP, RAFT, or ROMP. Prog. Polym. Sci..

[96] Heredia K.L., Maynard H.D. (2007). Synthesis of Protein–Polymer Conjugates. Org. Biomol. Chem..

[97] Staros J.V. (1982). N-Hydroxysulfosuccinimide Active Esters: Bis(N-Hydroxysulfosuccinimide) Esters of Two Dicarboxylic Acids Are Hydrophilic, Membrane-Impermeant, Protein Cross-Linkers. Biochemistry.

[98] Lewis M.R., Raubitschek A., Shively J.E. (1994). A Facile, Water-Soluble Method for Modification of Proteins with DOTA. Use of Elevated Temperature and Optimized pH To Achieve High Specific Activity and High Chelate Stability in Radiolabeled Immunoconjugates. Bioconjug. Chem..

[99] Suleiman E., Mayer J., Lehner E., Kohlhauser B., Katholnig A., Batzoni M., Damm D., Temchura V., Wagner A., Überla K. (2020). Conjugation of Native-Like HIV-1 Envelope Trimers onto Liposomes Using EDC/Sulfo-NHS Chemistry: Requirements and Limitations. Pharmaceutics.

[100] Gao Y., Sarode A., Kokoroskos N., Ukidve A., Zhao Z., Guo S., Flaumenhaft R., Gupta A.S., Saillant N., Mitragotri S. (2020). A Polymer-Based Systemic Hemostatic Agent. Sci. Adv..

[101] Kumai J., Sasagawa S., Horie M., Yui Y. (2021). A Novel Method for Polyacrylamide Gel Preparation Using N-Hydroxysuccinimide-Acrylamide Ester to Study Cell-Extracellular Matrix Mechanical Interactions. Front. Mater..

[102] Gee K.R., Archer E.A., Kang H.C. (1999). 4-Sulfotetrafluorophenyl (STP) Esters: New Water-Soluble Amine-Reactive Reagents for Labeling Biomolecules. Tetrahedron Lett..

[103] Charoenpattarapreeda J., Tan Y.S., Iegre J., Walsh S.J., Fowler E., Eapen R.S., Wu Y., Sore H.F., Verma C.S., Itzhaki L. (2019). Targeted Covalent Inhibitors of MDM2 Using Electrophile-Bearing Stapled Peptides. Chem. Commun..

[104] Poellmann M.J., Wagoner Johnson A.J. (2013). Characterizing and Patterning Polyacrylamide Substrates Functionalized with *N*-Hydroxysuccinimide. Cell. Mol. Bioeng..

[105] Trappmann B., Gautrot J.E., Connelly J.T., Strange D.G.T., Li Y., Oyen M.L., Cohen Stuart M.A., Boehm H., Li B., Vogel V. (2012). Extracellular-Matrix Tethering Regulates Stem-Cell Fate. Nat. Mater..

[106] Niu J., Page Z.A., Dolinski N.D., Anastasaki A., Hsueh A.T., Soh H.T., Hawker C.J. (2017). Rapid Visible Light-Mediated Controlled Aqueous Polymerization with in Situ Monitoring. ACS Macro Lett..

[107] Tsuji S., Aso Y., Ohara H., Tanaka T. (2019). Polymeric Water-Soluble Activated Esters: Synthesis of Polymer Backbones with Pendant N-Hydoxysulfosuccinimide Esters for Post-Polymerization Modification in Water. Polym. J..

[108] Tsuji S., Kobayashi K., Fujii T., Imoto H., Naka K., Aso Y., Ohara H., Tanaka T. (2022). Polymers with Pendant Water-Soluble Tetrafluorobenzene Sulfonic Acid Activated Esters: Synthesis, Stability, and Use for Glycopolymers in Water. Macromol. Chem. Phys..

[109] Tsuji S., Aso Y., Ohara H., Tanaka T. (2020). Aqueous Synthesis of Sialylglycopeptide-Grafted Glycopolymers with High Affinity for the Lectin and the Influenza Virus Hemagglutinin. J. Polym. Sci..

[110] Tsuji S., Aso Y., Tanaka T. (2023). Aqueous One-Pot and Oxygen-Tolerant Synthesis of Glycopolymers Using Polymer-Backbone-Bearing Water-Soluble Activated Esters. Chem. Lett..

[111] Jiang Z., Zhao H. (2023). Water-Soluble and Hydrolysis-Resistant Activated Ester Polymers: Synthesis, Characterization, and Application for Preparing Functional Polymers. J. Macromol. Sci. A.

[112] Breuck J.D., Streiber M., Ringleb M., Schröder D., Herzog D., Schubert U.S., Zechel S., Traeger A., Leiske M.N. (2024). Amino-Acid-Derived Anionic Polyacrylamides with Tailored Hydrophobicity–Physicochemical Properties and Cellular Interactions. ACS Polym. Au.

[113] Archer W.R., Gallagher C.M.B., Welborn V.V., Schulz M.D. (2022). Exploring the role of polymer hydrophobicity in polymer–metal binding thermodynamics. Phys. Chem. Chem. Phys..

[114] Illy N., Mongkhoun E. (2022). Thiolactone Chemistry, a Versatile Platform for Macromolecular Engineering. Polym. Chem..

[115] Reinicke S., Espeel P., Stamenović M.M., Du Prez F.E. (2013). One-Pot Double Modification of p(NIPAAm): A Tool for Designing Tailor-Made Multiresponsive Polymers. ACS Macro Lett..

[116] Wilkins L.E., Badi N., Du Prez F., Gibson M.I. (2018). Double-Modified Glycopolymers from Thiolactones to Modulate Lectin Selectivity and Affinity. ACS Macro Lett..

[117] Baysak E., Daglar O., Saim Gunay U., Hizal G., Tunca U., Durmaz H. (2018). Study on Post-Polymerization Modification of Ring-Opening Metathesis Polymers Involving Pendant Thiolactone Units. J. Polym. Sci. A Polym. Chem..

[118] Baysak E., Gunay U.S., Daglar O., Durmaz H. (2019). Synthesis and Post-Polymerization Modification of Polyester Containing Pendant Thiolactone Units. Eur. Polym. J..

[119] Reese C.M., Thompson B.J., Logan P.K., Stafford C.M., Blanton M., Patton D.L. (2019). Sequential and One-Pot Post-Polymerization Modification Reactions of Thiolactone-Containing Polymer Brushes. Polym. Chem..

[120] Misztalewska-Turkowicz I., Coutelier O., Destarac M. (2020). Two Pathways of Thiolactone Incorporation into Polyurethanes and Their One-Pot Double Postfunctionalization. Macromolecules.

[121] Kim J.S., Sirois A.R., Vazquez Cegla A.J., Jumai’an E., Murata N., Buck M.E., Moore S.J. (2019). Protein–Polymer Conjugates Synthesized Using Water-Soluble Azlactone-Functionalized Polymers Enable Receptor-Specific Cellular Uptake toward Targeted Drug Delivery. Bioconjug. Chem..

[122] Kakuchi R., Theato P. (2013). Three-Component Reactions for Post-Polymerization Modifications. ACS Macro Lett..

[123] Vong K.K.H., Maeda S., Tanaka K. (2016). Propargyl-Assisted Selective Amidation Applied in C-Terminal Glycine Peptide Conjugation. Chem. Eur. J..

[124] Vong K.K.H., Tsubokura K., Nakao Y., Tanei T., Noguchi S., Kitazume S., Taniguchi N., Tanaka K. (2017). Cancer Cell Targeting Driven by Selective Polyamine Reactivity with Glycine Propargyl Esters. Chem. Commun..

[125] Tanaka T., Iwamoto S., Aso Y. (2023). Accelerated Amidation of a Glycine Propargyl Ester on a Polymer and the Two-Way Use of One-Pot Double Postpolymerization Modification with Click Chemistry. Bull. Chem. Soc. Jpn..

